# Behavioral variability, elimination of responses, and delay-of-reinforcement gradients in SHR and WKY rats

**DOI:** 10.1186/1744-9081-3-60

**Published:** 2007-11-20

**Authors:** Espen B Johansen, Peter R Killeen, Terje Sagvolden

**Affiliations:** 1 Department of Physiology, Institute of Basic Medical Sciences, University of Oslo, Oslo, Norway; 2 Centre for Advanced Study at the Norwegian Academy of Science and Letters, Oslo, Norway; 3 Department of Psychology, Arizona State University, AZ, USA

## Abstract

**Background:**

Attention-deficit/hyperactivity disorder (ADHD) is characterized by a pattern of inattention, hyperactivity, and impulsivity that is cross-situational, persistent, and produces social and academic impairment. Research has shown that reinforcement processes are altered in ADHD. The dynamic developmental theory has suggested that a steepened delay-of-reinforcement gradient and deficient extinction of behavior produce behavioral symptoms of ADHD and increased behavioral variability.

**Method:**

The present study investigated behavioral variability and elimination of non-target responses during acquisition in an animal model of ADHD, the spontaneously hypertensive rat (SHR), using Wistar Kyoto (WKY) rats as controls. The study also aimed at providing a novel approach to measuring delay-of-reinforcement gradients in the SHR and the WKY strains. The animals were tested in a modified operant chamber presenting 20 response alternatives. Nose pokes in a target hole produced water according to fixed interval (FI) schedules of reinforcement, while nose pokes in the remaining 19 holes either had no consequences or produced a sound or a short flickering of the houselight. The stimulus-producing holes were included to test whether light and sound act as sensory reinforcers in SHR.

Data from the first six sessions testing FI 1 s were used for calculation of the initial distribution of responses. Additionally, Euclidean distance (measured from the center of each hole to the center of the target hole) and entropy (a measure of variability) were also calculated.

Delay-of-reinforcement gradients were calculated across sessions by dividing the fixed interval into epochs and determining how much reinforcement of responses in one epoch contributed to responding in the next interval.

**Results:**

Over the initial six sessions, behavior became clustered around the target hole. There was greater initial variability in SHR behavior, and slower elimination of inefficient responses compared to the WKY. There was little or no differential use of the stimulus-producing holes by either strain. For SHR, the reach of reinforcement (the delay-of-reinforcement gradient) was restricted to the preceding one second, whereas for WKY it extended about four times as far.

**Conclusion:**

The present findings support previous studies showing increased behavioral variability in SHR relative to WKY controls. A possibly related phenomenon may be the slowed elimination of non-operant nose pokes in SHR observed in the present study. The findings provide support for a steepened delay-of-reinforcement gradient in SHR as suggested in the dynamic developmental theory of ADHD. Altered reinforcement processes characterized by a steeper and shorter delay-of-reinforcement gradient may define an ADHD endophenotype.

## Background

Attention-deficit/hyperactivity disorder (ADHD) is a behavioral disorder characterized by a developmentally inappropriate pattern of inattention, hyperactivity, and impulsivity that is cross-situational, persistent, and produces social and academic impairment [1-3].

Motivational and environmental factors have an important influence on symptom development and expression in ADHD. Reinforcement contingencies in particular seem to affect behavior differently in ADHD than in controls [4-12].

The *dynamic developmental theory *of ADHD [13,14] suggests that dopamine hypofunction changes basic learning mechanisms by producing a narrower time window for the association of preceding stimuli, behavior, and its consequences. Further, it is suggested that this results in altered reinforcement processes in ADHD that are described by an abnormally steep and short delay-of-reinforcement gradient, and slower extinction of inefficient responses. Such deficits will result in a slower establishment of long integrated behavioral chains under proper stimulus control; partly due to slower chaining of behavioral elements and partly due to intrusion of inefficient and inadequate responses into the stream of behavior due to an inefficient extinction. The resulting behavior may be described as overactive, impulsive, inattentive, and variable [13-15].

The present study investigated predictions from the dynamic developmental theory in an animal model of ADHD. The spontaneously hypertensive rats (SHR) is a genetic model bred from its normotensive progenitor Wistar Kyoto rat (WKY), and has been validated as a model of ADHD [16-18]. SHR show the main behavioral characteristics of ADHD: Hyperactivity, impulsivity, inattention as well as increased behavioral variability [16-18]. Additionally, properties of the delay-of-reinforcement gradients in SHR and Wistar Kyoto (WKY) controls have previously been investigated; the behavioral changes in SHR being consistent with a steepened delay-of-reinforcement gradient compared to normal controls [16,18-22].

### Problem

**Figure 1:**
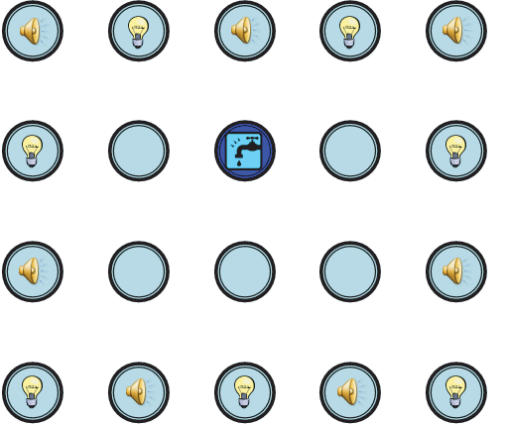
The layout of the panel in the hole-box is illustrated by the various symbols. The holes are designated numerically by the couplet (r, c), with the holes in the upper row as seen from the animal's working space designated (1, 1), (1, 2),...,(1, 5), row two (2.1), (2, 2),...,(2, 5). Nose pokes in the target hole (2, 3) produced water in a water cup on the opposite wall according to fixed interval schedules, while pokes in the other holes either had no consequences or produced a brief flickering of the houselight or a sound stimulus. The center of each hole was 4 cm from its nearest neighbor.

The present study investigated behavioral variability and elimination of non-target responses during acquisition in SHR and WKY controls. Further, the study aimed at providing a novel approach to measuring delay-of-reinforcement gradients in order to bring converging evidence to bear on the differences between the SHR and the WKY strains. The animals were tested in a modified operant chamber (*hole-box*) in which one wall contained 20 holes. Nose-pokes in the target hole produced water reinforcers according to fixed interval schedules of reinforcement while nose-pokes in the other holes either had no consequences, or produced a short flickering of the houselight or a brief sound stimulus (Figure 1). A previous study found a large effect of light-feedback on rate of lever pressing during extinction in SHR [23]. Hence, stimulus-producing holes were included in the present study to test whether light and sound act as sensory reinforcers in SHR. Properties of the delay-of-reinforcement gradients were investigated by dividing the fixed interval into epochs and calculating how much reinforcement of responses in an epoch affected responding in the next interval.

## Method

### Subjects

The subjects were eight male NIH-strain Spontaneously Hypertensive Rats (SHR) and eight NIH-strain Wistar-Kyoto (WKY) control rats. They were obtained from a commercial supplier (Møllegaard Breeding Centre, Denmark) at approximately 60 days of age, weighing 150–180 g. Subjects were housed four by four, 2 SHRs and 2 WKYs, in opaque plastic cages 35 × 26 × 16 cm (height) where they had free access to food (Beekay Feeds, Rat and Mouse Autoclavable Diet, B&K Universal Limited). The animal quarters were lit between 0800 and 2000 hours. The room temperature was kept at 20 ± 2°C and humidity at 55 ± 10%.

The study was approved by the National Animal Research Authority (NARA) of Norway, and was conducted in accordance with the laws and regulations controlling experiments/procedures in live animals in Norway.

### Apparatus

Three modified BRS/LVE Model RTC-022 Rodent Test Cages (A, B, C) located within standard BRS/LVE (SEC-002) outer housings, and one modified LeHigh Valley Model 1417 Rodent Test Cage (D) within a standard LeHigh Valley Model 1417 Small Environment outer housing were used as experimental chambers. The rat's working space was 26.5 × 25.0 × 26.5 (height) cm. There was no lever, but one wall was a metal panel 25.9 × 26.5 cm containing 20 2.0 cm diameter holes, designed as response locations for nose poking. The holes were arranged in five parallel columns with four holes in each column, and were designated numerically by the couplet (r, c), with the holes in the upper row as seen from the animal's working space designated (1, 1), (1, 2),...,(1, 5) (Figure 1).

The center-to-center distance between holes was 4.0 cm for both rows and columns. The bottom row was located 2.0 cm above the floor, and the top row 12.5 cm below the ceiling of the cage. Poking deeper than 8.5 mm into the hole was detected by photocells in each hole. Activation of holes (2, 1), (4, 1), (1, 2), (4, 3), (1, 4), (2, 5), and (4, 5) flickered the 15 W houselight for as long as the animal was in the hole. This function is represented by the lamps in the diagram of Figure 1. Activation of holes (1, 1), (3, 1), (4, 2), (1, 3), (4, 4), (1, 5), and (3, 5) generated a brief 95 dBA, 3 kHz tone (cage A, B, C), or a 95 dBA, 4.9 kHz tone (cage D) from an amplifier (Sonalert) placed inside the test chamber. This symmetric distribution of "light-" and "sound-" holes permitted to check for preferences. Activation of the holes (2, 2), (3, 2), (3, 3), (2, 4), and (3, 4) close to the target hole (2, 3) produced no stimuli. Activation of the target hole (2, 3) was immediately followed by 0.01 ml tap water delivered with a loud click by a liquid dipper on the opposite cage wall. The liquid dippers were of models BRS/LVE Model SLD-002 (cage A, B and C) and LeHigh Valley Model 1351 (cage D). The 0.01 ml water cup on the liquid dipper protruded through a hole within the recessed cup shield. The water cup was positioned 0.5 cm above floor level 0.5 cm (depth) into the opening and the shield was 3 cm in diameter and 2 cm deep. A photocell positioned 0.5 cm into the wall of the cup shield recorded all visits during experimental sessions.

A 15 W houselight located in the center of the ceiling illuminated the cage. White masking noise was provided by the ventilation fans (65 dBA). Sessions were signaled by onset of the houselight and the white masking noise. All photocell beam breaks were recorded by the computer with 55 ms accuracy. Complete records of all hole-visits were kept.

### Procedure

The experiment was run 5 days a week for most of the experimental period. The final sessions were run 7 days a week. All sessions were run between 1530 and 1900 hours. The duration of each session varied to some extent due to differences in the total number of reinforcers programmed for the session, schedule, and time each rat needed to complete the schedule (see Table 1). Due to low response rates in some animals, other animals ended the session earlier but remained in the darkened chamber until all in their squad had completed their session. Each subject was always run in the same experimental chamber. All four rats housed together in a cage were run at the same time every day to allow a constant water deprivation. The animals were deprived of water for 22.5 h before each session. Immediately following the sessions, the animals were returned to their home cages where tap water was available ad lib. for 30 min from multiple water bottles in each cage.

**Table 1:** Summary of the experimental procedure. FI: fixed interval schedule of reinforcement

Session number	Schedule	No. of reinforcers
1 – 6	FI 1 s^1^	20
7 – 8	FI 15 s	20
9 – 11	FI 30 s	20
12 – 13	FI 60 s	15
14 – 15	FI 120 s	10
16	FI 200 s	10
17 – 54	FI 300 s^2^	6

Note. – ^1 ^Used for analyses of entropy, and initial Euclidean distance (Figures 3, 4 and 5).

^2 ^All sessions testing FI < 300s and the last 21 sessions testing FI 300 s were used for analyses of influence functions and overall Euclidean distance (Figures 5 and 6).

### Response acquisition

On arrival, all animals were registered, marked, assigned to four separate groups for housing, and subsequently handled. Habituation to the experimental chamber and magazine training were conducted during the four sessions immediately preceding response shaping. During magazine training, all holes in the panel were covered, and water was available from the water cup on a random time (RT) 10 s schedule (two sessions) and on a RT 20 s schedule (two sessions). Such schedules provide water at random time intervals independent of the rat's behavior. By the fourth magazine training session, all animals reliably collected the reinforcers when available.

### Shaping

Only the target hole (2, 3) was available during response shaping. Nose poking into the target hole was hand-shaped by the method of successive approximations (two sessions).

### The fixed-interval reinforcement schedule

The fixed-interval (FI) schedule delivers a reinforcer for a correct response that occurs after a fixed time since the previous reinforcer. The reinforced operant was the activation of the photocell in the target hole (2, 3). The sound from the electromagnet operating the water cup signaled the availability of water. Holes other than the target hole were covered until the subjects reliably emitted enough appropriate responses to produce 20 reinforcers programmed on a FI 1 s schedule in every session. Then all holes were uncovered. The first session with all holes uncovered is numbered as Session 1, and marks the start of the data set to be reported here. A gradual increase in FI value, and a compensatory decrease in the number of reinforcers available, proceeded until session 17 when the FI 300-s schedule was introduced. The number of available reinforcers was 20 during FI 1s and decreased to 6 during FI 300 s (Table 1). The gradually increasing FI values were intended simply to ensure a smooth transition to the longest schedule, FI 300 s, which was used throughout the rest of the study.

### Data analyses

Data from the first six sessions testing FI 1 s were used for calculation of the initial distribution of responses, Euclidean distance, and entropy. These sessions were selected to capture behavior as the animals were acquiring a new repertoire. The data analyzed were rate of responding in the four types of holes – light, sound, neutral, and water – and rate of investigating the water tray. These are reported as responses per second. To avoid redundant counts for sniffing at holes or tray, no activity was registered until at least 120 ms had elapsed since the previous registered response. Delay-of-reinforcement gradients were calculated based on data from all sessions testing FI < 300 s and the last 21 sessions testing FI 300 s. The following measures were calculated:

#### Distance

The Euclidean distance was measured from the center of each hole to the center of the target hole (2, 3).

#### Entropy

Entropy is a measure of the variability of responding. It is calculated as the sum of the probabilities of visiting each hole multiplied by the logarithms of those probabilities: *U *= -Σ*plog*_*2*_*(p)*. Probabilities were calculated as relative frequencies over the blocks of 100 events (nose pokes, visits to the water cup, and reinforcers). Entropy does not take into account the order of visiting holes, or their distance from one another. It is measured in bits, and ranges from 0, in case every response is to the same hole, to 4.32, in case responses are distributed to each of the 20 holes with equal probability.

#### Delay-of-reinforcement gradient

A reinforcer acts on responses that occurred immediately before its receipt, and to a lesser extent on those that occurred at some temporal remove. The decrease in efficacy of reinforcement as a function of the time elapsing between a response and the reinforcer is called the delay-of-reinforcement gradient. It presumably occurs because the memory of the response on which the reinforcer acts (the response trace) decays over time. Here, the gradient is calculated from all sessions testing FI < 300s and the last 21 sessions testing FI 300 by (a) noting which responses occur in various epochs before a reinforcer is delivered; the epochs are bins of increasing size, centered at 0.15, 0.64, 1.5, 2.6, 4.3, 6.6, 9.6, 14, 20, 28, 40, 55, 73, 95, and 100 s. These steps approximately equated the number of observations for each epoch, while providing both a relatively fine scale at the steepest portion of the gradient, and stability of estimates as the distance increased from the following reinforcer. Whenever such a response is recorded, a counter of opportunities for that epoch is incremented. (b) A counter of the number of times that each such response occurs *any *time in the next interval is incremented. (c) The number of observations of a repeated response divided by the number of opportunities for observing such a response gives the relative frequency with which a response is observed following reinforcement as a function of its proximity to reinforcement in the prior interval. These calculations, modeled after [24], provide a measure of the differential emission in the future of behavior that was reinforced at different temporal removes in the past. The measure, a relative frequency, is independent of overall rate of responding.

## Results

### Use of holes

**Figure 2:**
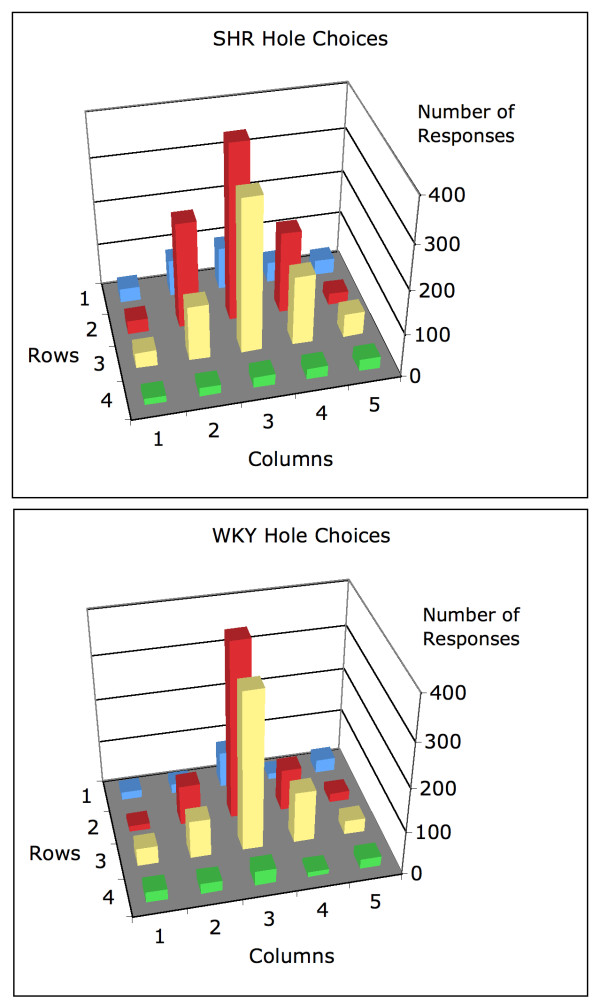
The total number of hole entries made by rats during the first 6 sessions of FI 1 s. The graphs are truncated at 400 responses. The number of target hole (2, 3) responses was approximately 1200 for both strains.

Figure 2 shows the total number of hole entries over all animals during the first six sessions of FI 1 s. These graphs are truncated at 400 responses, with the number of target hole responses rising to approximately 1200 for each strain of rat. It is clear that the most frequently entered hole after the target hole is the one just below it (3, 3), and that hole use in general conformed to a simple spatial generalization gradient. Although the two graphs look similar, a Chi-Square test shows them to be significantly different (*χ*^2^(19, 5253) = 218, p < .01, p_rep _> .99), the difference consisting of a flatter spatial generalization for SHR.

The average response rate of the WKY rats over these sessions was 6.74 responses per minute, with about half of those responses to the target hole (3.70 responses per minute). The SHR responded almost twice as fast (11.1 responses/minute), with about a third of their responses to the target hole (4.42 responses/min). The higher overall response rate is due in large part to the greater incidence of responses to neutral holes, as those did not lead to operation of the water dipper, and did not occasion the animal's trip to the dipper and the start of a new trial.

### Initial learning

**Figure 3:**
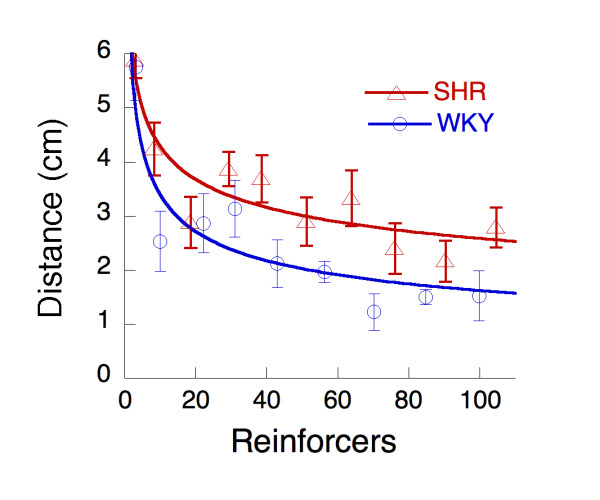
The average distance of a hole-poke from the target hole, per 100 events (hole-pokes, visits to the water cup, and reinforcers) as a function of the number of reinforcers received during the first 6 sessions of FI 1 s. The acquisition curves are drawn by Equation 1.

Upon initial exposure to all holes, all rats probed most of the holes. Over the course of the first 6 sessions of FI 1 s with all holes available, the distribution of responses narrowed, becoming both more focused on the target hole, and becoming less variable overall. This is visible in Figure 3, where the average distance of hole-pokes from the target hole is plotted as a function of number of reinforcers (*n*). The curves are simple power functions, which are often used to describe learning curves:

d¯n=d1n−β
 MathType@MTEF@5@5@+=feaafiart1ev1aaatCvAUfKttLearuWrP9MDH5MBPbIqV92AaeXatLxBI9gBaebbnrfifHhDYfgasaacPC6xNi=xI8qiVKYPFjYdHaVhbbf9v8qqaqFr0xc9vqFj0dXdbba91qpepeI8k8fiI+fsY=rqGqVepae9pg0db9vqaiVgFr0xfr=xfr=xc9adbaqaaeGacaGaaiaabeqaaeqabiWaaaGcbaGafmizaqMbaebadaWgaaWcbaGaemOBa4gabeaakiabg2da9iabdsgaKnaaBaaaleaacqaIXaqmaeqaaOGaemOBa42aaWbaaSqabeaacqGHsisliiGacqWFYoGyaaaaaa@36C9@

where d¯n
 MathType@MTEF@5@5@+=feaafiart1ev1aaatCvAUfKttLearuWrP9MDH5MBPbIqV92AaeXatLxBI9gBaebbnrfifHhDYfgasaacPC6xNi=xH8viVGI8Gi=hEeeu0xXdbba9frFj0xb9qqpG0dXdb9aspeI8k8fiI+fsY=rqGqVepae9pg0db9vqaiVgFr0xfr=xfr=xc9adbaqaaeGacaGaaiaabeqaaeqabiWaaaGcbaGafmizaqMbaebadaWgaaWcbaGaemOBa4gabeaaaaa@2ECD@ is distance in cm around the time of the *n*^th ^reinforcer, the parameter *d*_1 _is the average distance projected to the time of the first reinforcer, and *β *is the rate of learning. Both strains start from an average distance of *d*_1 _= 7.1 cm, but the rate of learning is faster for WKY (*β *= 0.32) than for SHR (*β *= 0.22).

**Figure 4:**
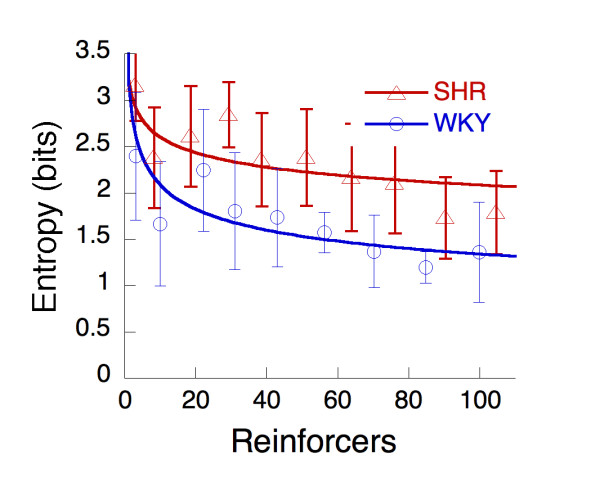
The average entropy of hole-poking, per 100 events (hole-poke, dipper approach, and reinforcement) as a function of the number of reinforcers received during the first 6 sessions of FI 1 s. The acquisition curves are drawn by Equation 1 acting on entropy (*U *= -Σ*plog*_*2*_*(p))*, the sum of the logarithms of the probabilities of visiting each hole weighted by that probability.

In Figure 4, response variability, expressed as entropy, is plotted as a function of reinforcers during acquisition. For both strains, the decreasing variability is described by Equation 1. The SHR start slightly more variable (*U *= 3.7) and may focus more slowly (*β *= 0.13) than WKY (*U *= 3.0, *β *= 0.18). Given the width of the error bars, however, all that can be said with confidence is that the entropy curve for the SHR lies above that for the WKY. The reduction in variability of responding was largely due to the convergence of behavior onto the operant target hole.

The holes around the periphery provided additional stimulation which seemed more attractive than the neutral holes. Figure 2 shows, however, that any additional attractiveness of the stimulus holes may be attributed to their spatial layout, not their sensory consequences.

### Delay-of-reinforcement gradients

**Figure 5:**
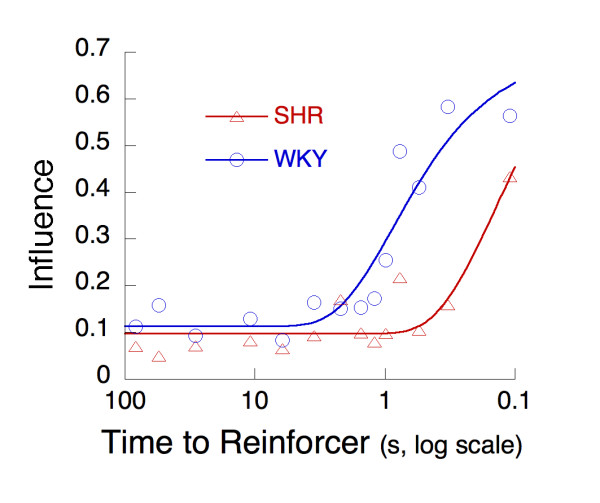
Delay-of-reinforcement gradients calculated from all sessions testing FI < 300s and the last 21 sessions testing FI 300 and pooled over eight SHR and eight WKY rats. The influence of a reinforcer is measured as the probability that a response at a given remove from the reinforcer would reappear somewhere in the next interval. For these data, the curves start equally high for SHR and WKY (*c + b *equals 0.617 and 0.647, respectively) while rate of decrease is faster for SHR (*λ *= 0.63 s^-1^) than WKY (*λ *= 0.38 s^-1^).

To what extent can a reinforcer increase the probability of not only the response that immediately preceded it, but also the probability of other, earlier responses? Figure 5 shows real delay-of-reinforcement gradients calculated from all sessions testing FI < 300s and the last 21 sessions testing FI 300 in the manner detailed in the procedure section. They are shown on a logarithmic *x*-axis to highlight the time intervals closest to reinforcement. The data are pooled across all animals within a strain. The curves through the data are exponential processes, such as those represented in Equation 2, where the parameter *c *gives the height of the gradient above its asymptotic level, *b*, at the time of reinforcement (*t *= 0). The parameter lambda gives the rate of decrease in the gradient as a function of the time between a response and the ensuing reinforcer. The additive constant *b *measures the asymptotic probability of emitting the same response on succeeding trials.

*Influence *= *ce*^-*λt *^+ *b*

Whereas Figure 5 gives a representative summary of the delay-of-reinforcement gradients, a more precise account is obtained by fitting Equation 2 to the data of individual rats, weighting each time bin by the number of opportunities for observing a repetition it contains. The results of this analysis are presented in Table 2 where their contribution to the average was weighted by the average goodness of fit of the model to their data. There is no strain difference in the immediate impact of the reinforcer (measured by the coefficient *c*), or in the asymptotic probability (measured by the additive constant *b*), but there is an obvious difference in the impact of the reinforcer on the responses preceding it: For SHR, the reach of the reinforcer is restricted to the preceding one second, whereas for WKY it extends almost four times as far.

**Table 2:** Parameters describing the delay-of-reinforcement gradients

Strain	Average Parameters (SEM)		
	*c*	*λ*	*b*
SHR	0.506 (0.048)	3.89 (0.63)	0.111 (0.011)
WKY	0.528 (0.039)	0.95 (0.38)	0.119 (0.025)

Note. – The average parameters of the equation. *Influence *= *ce*^-*λt *^+ *b *representing the delay-of-reinforcement gradients for every rat. The parameters of each rat were weighted by the goodness of fit of that model to their data. The semi-interquartile ranges of the parameters are in parentheses. All sessions testing FI< 300s and the last 21 sessions testing FI 300 s were used for analyses of influence functions.

An alternate analysis that excludes the water hole responses yields flatter gradients with WKY lying above SHR, thus showing their generally greater susceptibility to reinforcement.

### Subsequent performance

**Figure 6:**
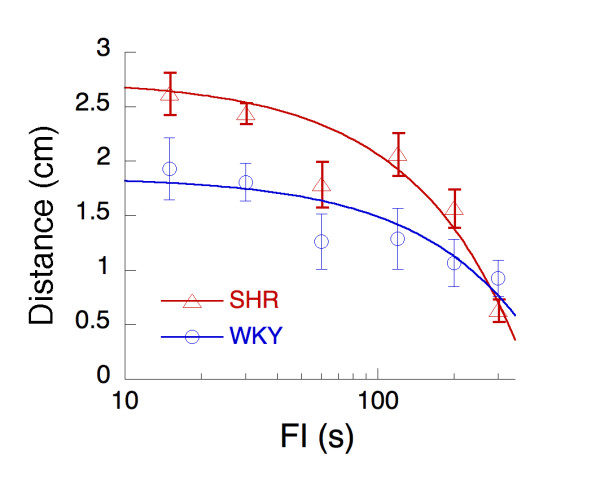
The average distance from the target hole as a function of successive FI values calculated for all sessions testing FI < 300s and the last 21 sessions testing FI 300. An approximately linear convergence on the central water hole draws concave functions on these coordinates.

The allocation of responses continued to converge on the target hole with ongoing experimentation. Figure 6 shows that the SHR continued to explore other holes more than the WKY up to the longest FI, where both rate of responding and distance from the target hole decreased substantially.

## Discussion

The dynamic developmental theory of ADHD suggests that dopamine hypofunction produces a narrower time window for associating preceding stimuli, behavior, and its consequences, behaviorally described as a steeper and shorter delay-of-reinforcement gradient. The theory also suggests that dopamine hypofunction causes slowed extinction of inadequate behavior. These changes in basic learning mechanisms are suggested to produce symptoms of ADHD and increased behavioral variability [13]. In a strictly behavior-analytic sense, delay-of-reinforcement gradients can not be considered explanatory (it would be a category mistake to use observations of behavior to explain behavior). However, the theory is also based on neurobiological evidence and knowledge of how reinforcement processes are linked to dopamine function. Hence, evidence of dopamine dysfunction in ADHD [13] combined with findings on how dopamine modulates neuronal activity and plasticity is an explanation of why effects of reinforcers are altered in ADHD and how dopamine dysfunction translates into what in behavioral terms can be described by a steepened delay-of-reinforcement gradient.

The present study examined behavioral variability, elimination of non-operant responses, and properties of the delay-of-reinforcement gradient in an animal model of ADHD, the spontaneously hypertensive rat (SHR). SHR and Wistar Kyoto (WKY) controls were tested in operant chambers presenting 20 response alternatives (holes in the wall). Nose-pokes in a target hole produced water reinforcers according to fixed interval schedules of reinforcement, while nose-pokes in the remaining 19 holes either had no consequences, or produced a brief sound or a short flickering of the houselight (Figure 1). The stimulus-producing holes were included in the present study to test whether light and sound act as sensory reinforcers in SHR [23].

### Behavioral variability and elimination of non-target nose pokes

The dynamic developmental theory of ADHD states that a combination of a short delay-of-reinforcement gradient, which will hamper the establishment of long integrated behavioral chains, and a deficient extinction process will result in increased behavioral variability [13,20,25,26]. In the present study, entropy and Euclidean distance were calculated for responding across the response alternatives during the first six sessions following response shaping and used as measures of intra-individual behavioral variability. The results provide support for the suggestion of increased behavioral variability in SHR compared to WKY controls. Response variability, expressed by entropy, started out higher in SHR than in the controls (Figure 4). Variability decreased as training progressed, largely due to the convergence of behavior onto the operant target hole (Figure 3), consistent with Antonitis' early work showing that variability of nose pokes in rats decreased as a function of number of reinforcers [27]. The rate of convergence was faster for the WKY, markedly in focusing of distance (Figure 3) but slightly in decrease in variability (Figure 4). More holes were explored by SHR than WKY up to the longest FI where both rate of responding and distance from the target hole decreased substantially (Figure 6).

The present data do not show that light and sound have reinforcing properties in SHR [23]. There was little or no differential use of the stimulus-producing holes by either strain, as seen in Figure 2. The flatter generalization gradient in SHR relative to WKY controls (Figure 2) might be interpreted as a spatial discrimination problem in SHR, possibly producing more variable responding. Using a spatial memory maze, Low and co-workers [28] also found more variable behavior in SHR compared to WKY controls. However, while a possible spatial discrimination problem in SHR cannot be ruled out, studies have shown that SHR behave more variable in lever-pressing tasks with only two response alternatives [18,29,30] and independently of the reinforcement contingencies [31], suggesting that behavioral variability in SHR has other origins.

Focusing of behavior onto the operant hole and decrease in variability were retarded for SHR in the present study, consistent with the dynamic developmental theory which predicts retarded elimination of ineffective responses in SHR [13,20,25,26]. The theory predicts that behavior in both strains starts out variable and then become organized as a function of learning, but more slowly in SHR. However, the present findings show that behavioral variability, as expressed by entropy (Figure 4), also seems to *start *out higher in SHR relative to WKY controls. Hence, while the slower decrease in behavioral variability in SHR may be related to deficient learning processes, initial variability may be unrelated to reinforcement processes, or could be linked to the general behavior-inducing effects of the presence of reinforcers (below).

### The delay-of-reinforcement gradient

The effect of a reinforcer on a response decreases as the time interval between the response and reinforcer delivery is lengthened [32], which also applies to responding during FI schedules [33]. Here, delay-of-reinforcement gradients were calculated for SHR and controls by dividing the fixed interval into epochs and counting the various responses within each epoch that recurred in the next interval. As the time to reinforcement varied across the epochs, we could calculate how this time interval affected reinforcer effectiveness: How much did reinforcement of a response in a particular epoch contribute to responding in the next fixed interval trial. The results support the suggested steepened and shorter delay-of-reinforcement gradient in SHR compared to WKY [18]. The impact of a reinforcer on the preceding responses is restricted to the preceding one second in SHR whereas for WKY it extends nearly four times as far (Figure 5). No significant strain differences in the immediate impact of a reinforcer (the intercept at a delay of 0 s) or in the asymptotic probability were found.

The finding of a steepened delay-of-reinforcement gradient in SHR relative to WKY controls is consistent with previous studies using both water reinforcers and intra-cranial self-stimulation (ICSS) [16,18-22]. In a study by Evenden and Meyerson [34], SHR and WKY controls were required to make a minimum number of consecutive presses on one lever before switching to another lever for a final lever-press to produce the reinforcer (fixed consecutive number schedule of reinforcement, FCN). They found that SHR made fewer long chains on the FCN schedule, consistent with the steepened delay-of-reinforcement gradient seen in Figure 5[34]. Hand and co-workers [35] used a 15-s resetting reinforcer delay procedure and demonstrated retarded response acquisition in SHR compared to WKY controls. Consistent with a steepened delay-of-reinforcement gradient, SHR exhibited lower response rates and earned fewer reinforcers during reinforcer delay, but responded more during immediate reinforcer delivery than WKY controls [35].

The delay-of-reinforcement gradients found in the present study are short given Hand and co-workers' findings [35] and the demonstrated ability of rats to learn new responses at delays of up to 30 s (e.g., [36,37]). However, there are many competing responses in the current experiments, and the rapid delivery of the reinforcer and the change in the constellation of responses as a function of learning all contribute to this shortening of the gradient. This difference in gradients could be a by-product of the hyperactivity of the SHR, which fills the delay interval with more, and more diverse, behaviors than it does for the WKY, and depresses the gradient by making diverse, rather than target hole responses, more attractive to SHR. However, such an account does not explain why hyperactivity in SHR develops [18,19]. The dynamic developmental theory explains the increase in activity as the combined effect of selective reinforcement of short interresponse times and deficient extinction [13,14]. The theory suggests that the delay-of-reinforcement gradient is steeper and shorter, and initially lower, in SHR compared to WKY controls. Although the effect of reinforcers is lower in SHR, the theory suggests that a steepened delay-of-reinforcement gradient means that mainly short interresponse times are reinforced, leading to an increased activity level. Deficient extinction would add to activity level by inadequate pruning of ineffective behavior [13,14].

The delay gradients calculated in the present study are independent of overall rates of responding. Thus, a second possibility is that SHR hyperactivity is produced by a delay-of-reinforcement gradient that is steeper and shorter, but starts higher, in SHR compared to WKY controls [15]. This means that a reinforcer immediately following a response has a higher effect in SHR than in WKY controls, producing a higher rate of responding. However, our data show that number of pokes in the target hole was similar in the two strains (approximately 1200; Figure 2 is truncated at 400 responses), suggesting that the effect of immediate reinforcers is similar in SHR and WKY (Figure 5). Still, SHR did have a higher rate of responses than WKY controls, the strain difference mainly produced by the higher rate of visits to the neutral holes in SHR (Figure 2). If the higher rate of visits to the neutral holes in SHR is caused by a genuine discrimination problem, it will cause problems for how we calculated the delay-of-reinforcement gradients because the underlying logic is that a reinforced response will be repeated in the same hole in the next fixed interval. If the exact repetition of a response is problematic for SHR due to a discrimination problem, the implication is that the impact of an immediate reinforcer may be higher in SHR than in WKY controls.

However, a third possibility is that the behavior-inducing effects of reinforcers contribute to SHR overactivity. Presence of reinforcers increases general activity [38], a process that may be linked to physiological arousal and noradrenergic function [39]. Reinforcer presence may produce more arousal and induce more behavior in SHR than in controls, consistent with studies showing elevated noradrenergic levels [40,41] and increased sympathetic nervous system activity in SHR compared to WKY controls [42]. The higher level of induced general behavior in SHR may show up as higher initial behavioral variability compared to WKY controls. Additionally, effects of periodic reinforcers seem to cumulate [38] predicting that SHR hyperactivity will develop as a function of exposure to reinforcers, consistent with previous observations [17-19].

Theories of reinforcement have suggested that behavioral arousal and the selective strengthening of behavior with favorable outcomes are independent components of the reinforcement process (e.g. [43,44]). Hence, while it is conceivable that the steepened delay-of-reinforcement gradient calculated for SHR in the present study is an artifact of the overactivity and increased behavioral variability in SHR, both behavioral arousal and the selective strengthening of behavior could be changed in SHR. Indeed, studies manipulating reinforcer delay while keeping reinforcer frequency relatively constant have shown that SHR behavior is more sensitive to reinforcer delay than WKY controls [19,22,35]. This suggests that a steepened delay-of-reinforcement gradient in SHR is not secondary to behavioral arousal but is making an independent contribution to behavior in SHR, consistent with studies showing *both *dopamine and noradrenaline changes in SHR [17,41]. A steepened delay-of-reinforcement gradient in SHR would imply slower and less efficient response differentiation than in normal controls. The combination of increased behavioral arousal produced by reinforcers and a steepened delay-of-reinforcement gradient in SHR is consistent with present findings: Increased behavioral variability both initially as well as with continued training, slowed response differentiation (Figures 3 and 4), and a steepened delay-of-reinforcement gradient (Figure 5). Additionally, it also predicts the previously observed development of hyperactivity as a function exposure to reinforcers [17-19], the increased sensitivity to reinforcer delay in SHR [19,22,35], and the higher rate of responses with short interresponse times consistently observed in SHR [16,18]. However, although the suggestion of increased behavioral arousal produced by reinforcers combined with a steepened delay-of-reinforcement gradient in SHR seems to integrate several findings, and is supported by some evidence, it needs further testing.

## Conclusion

The dynamic developmental theory of ADHD suggests that reinforcement and extinction processes are altered in ADHD due to dopamine dysfunction, and suggests that the altered reinforcement processes behaviorally can be described as a steeper and shorter delay-of-reinforcement gradient in ADHD compared to normal controls. The theory has outlined how a steeper delay-of-reinforcement gradient and deficient extinction can produce the main behavioral symptoms of ADHD: Inattention, hyperactivity, and impulsivity, in addition to increased behavioral variability that seems to be a characteristic of ADHD.

The results support the hypothesized steeper and shorter delay-of-reinforcement gradient in the animal model, and provided some support for the increased behavioral variability suggested by the dynamic developmental theory of ADHD: Behavior was more variable initially, decreased somewhat slower, and settled into comparable levels of variability only with extended training.

In conclusion, altered reinforcement processes may be a characteristic of an ADHD phenotype. Investigations on how reinforces work in ADHD may provide new insights into symptom development, sources of behavioral variability, and how behavior most efficiently can be focused onto adequate behavior favored by parents and teachers. This knowledge may lead to the development of more optimal interventions and treatment strategies.

## Competing interests

The author(s) declare that they have no competing interests.

## Authors' contributions

TS designed study and supervised the experiment. EBJ had the main responsibility of writing the manuscript. PRK performed the mathematics and wrote parts of the manuscript. All authors were involved in interpreting the data, read and approved the final manuscript.
